# The Molecular Design of a Macrocycle Descaling Agent Based on Azacrown and the Mechanism of Barium Sulfate Scale Removal

**DOI:** 10.3390/molecules29215167

**Published:** 2024-10-31

**Authors:** Da Wu, Dexin Liu, Minghua Shi, Jiaqiang Wang, Han Zhao, Yeliang Dong

**Affiliations:** 1 State Key Laboratory of Deep Oil and Gas, China University of Petroleum (East China), Qingdao 266580, China; 2 School of Petroleum Engineering, China University of Petroleum (East China), Qingdao 266580, China

**Keywords:** density functional theory, adsorption energy, macrocycle, barium sulfate

## Abstract

The formation of barium sulfate scale is a persistent and formidable challenge across various industrial processes. In order to effectively mitigate this problem, this study proposed the development of an innovative azacrown ether-based macrocycle descaling agent. Using density functional theory, an in-depth analysis of the surface energy of different barium sulfate crystal facets was carried out, together with a detailed investigation into the adsorption properties of the functional groups on the (001) surface. A further comprehensive investigation was carried out to determine how changes in the nitrogen and oxygen atoms in the crown ether framework influence its adsorption affinity to barium ions. In addition, a detailed analysis was carried out to elucidate the molecular interactions between crown ethers with pyridine carboxylic acid side chains and barium sulfate. The newly developed decalcifying macrocycle descaling agent exhibited superior adsorption performance, achieving an adsorption energy for barium ions approximately −4.1512 ev higher than that of conventional DTPA decalcifiers. This remarkable improvement is mainly attributed to the pivotal role of electrostatic forces in the coordination process between the macrocycle descaling agent and barium ions, with an electrostatic potential value reaching −143.37 kcal/mol. This discovery not only introduces a novel approach to the removal of barium sulfate scale but also highlights the significant potential of macrocycle chemistry in industrial applications.

## 1. Introduction

Barium sulfate (BaSO_4_) scaling is a common challenge in industrial systems, particularly in the oil and gas sector [1,2,3]. Due to its remarkably low solubility in aqueous environments, barium sulfate is preferentially deposited onto equipment surfaces, leading to blockages and reduced operational efficiency [4,5]. Conventional scale removers often pose environmental hazards and have suboptimal efficacy. For instance, acidic descaling agents can cause corrosion of equipment and may fail to completely remove entrenched layers of scale. Such limitations highlight the urgent need for innovative barium sulfate scale removers, yet there remains a lack of effort focused on the design and development of such agents. Most of the current research is concentrated on the formulation of chelating agents [6,7,8], including DTPA or EDTA, making the design of novel chelating agents increasingly imperative.

Azacrown ethers have attracted considerable attention in supramolecular chemistry due to their distinctive cyclic structures and their ability to interact highly specifically with metal ions [9,10,11]. The synthesis and application of azacrown ethers have always been central areas of research in chemistry, particularly in the fields of molecular recognition and catalysis. The incorporation of water-soluble groups into the azacrown ether ring allows efficient synthesis of water-soluble azacrown ether macrocyclic host molecules. These host molecules recognize viologen salts primarily through the synergistic effects of multiple weak interactions, including electrostatic and π-π interactions. The design and synthesis of water-soluble azacrown ether macrocyclic host molecules with multiple recognition sites and high complexation constants are major research directions in this field [12,13,14].

In response to this challenge, we have developed a macrocycle scale remover derived from azacrown ethers. Azacrown ethers were chosen for their distinctive cyclic structure and their ability to interact specifically with metal ions. Using density functional theory (DFT), we investigated the surface energy of different facets of barium sulfate crystals. We observed that changes in the nitrogen (N) and oxygen (O) atoms within the crown ether significantly influenced the adsorption of barium ions. The incorporation of this side chain is intended to enhance the interaction with barium sulfate while minimizing metal surface corrosion. Compared to conventional chelating agents such as DTPA, our macrocycle scale remover demonstrates superior selectivity and affinity. Through precise design and molecular simulation, we aim to develop novel macrocycle scale removers that provide an innovative strategy for industrial-scale cleaning. With this strategy, we aim to provide chemists with a novel tool for the efficient and timely synthesis of macrocycle scale removers. This will enable more efficient and environmentally sustainable scale inhibition in industrial processes.

## 2. Simulation Methods

Based on data retrieved from the Findit database, barium sulfate crystallizes in the orthorhombic space group PNMA (#62), characterized by unit cell parameters a = 8.8842 Å, b = 5.4559 Å, and c = 57.1569 Å, with α = β = γ = 90.000°. The surface energy and adsorption energy for functional groups of barium ions on the primary crystal facets were accurately calculated using the Dmol3 module in Materials Studio. The calculation accuracy was set to ‘Fine’, ensuring an energy convergence threshold of 1.0 × 10^−5^ Ha, a maximum force convergence of 0.002 Ha/Å, a maximum displacement convergence of 0.005 Å, and a maximum of 500 iterative steps. To reduce the computational effort, the effective core potential (ECP) [15,16] was used to substitute the core electrons, while the atomic orbitals were defined using the DNP 3.5 basis set in conjunction with the GGA (Generalized Gradient Approximation)/PBE (Perdew–Burke–Ernzerhof) functional [17,18]. The functional adsorption experiment used the (001) crystal plane of barium sulfate (BaSO_4_) as a substrate. This crystal plane is characterized by specific lattice parameters, which are a = 17.768 Å, b = 16.368 Å, and c = 25.042 Å. In the simulation, the calculations of the electrostatic and van der Waals forces were based on the COMPASSII force field algorithm, with parameter selection and optimization performed using the Smart algorithm.

In the calculated simulation of the adsorption of barium ions by azacrown ethers and DTPA, we used a one-to-one adsorption method, i.e., each molecule corresponded to one barium ion. We ensured the standard agreement of the parameters calculated with the parameters for functional group adsorption. In addition, we used the Mulliken charge analysis method to calculate the charge transfer for each molecule with barium ions before and after adsorption.

In the molecular dynamics simulation, the size of the box used was a = 26.6526 Å, b = 27.2795 Å, and c = 65.0 Å, with 4 layers of barium sulfate (the top layer was relaxed, and the bottom three layers were fixed), filled with 500 molecules of water and 1 molecule of descaling. The precision of the calculation was Fine, and the NVT system was selected. The temperature was 298 K, the step size was 1 fs, the total time length was 500 ps, and 1 frame was output every 5000 steps.

## 3. Results and Discussion

### 3.1. Surface Energy Calculation

**Figure 1:**
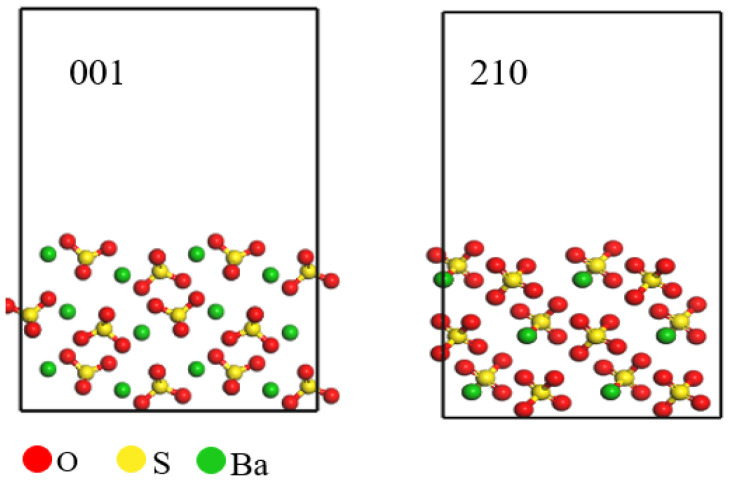
Barium sulfate 001 and 210 crystal faces.

In conducting a theoretical study of barium sulfate, clarification of interface theory plays a vital role in the overall results of the study. The primary goal of this study is to determine the interfacial energy. First, we need to confirm the interfacial properties of barium sulfate to ensure the accuracy and validity of this study. Surface energy quantifies the relative energetic state of a material’s surface compared to its bulk interior, thereby indicating the stability of the surface. A lower surface energy corresponds to a more stable surface. As illustrated in Table 1, the 001 crystal face of barium sulfate has the lowest surface energy of 0.0309 J·m^−2^, indicating that this face has lower energy unsaturation and enhanced structural stability. The surface energy of the 210 crystal face of barium sulfate is 0.0327 J·m^−2^, slightly higher than that of the 001 face but still indicating considerable structural stability. It is concluded that the 001 and 210 crystal faces are the most stable faces of barium sulfate [19,20,21], which is consistent with conclusions found in the literature [22]. Furthermore, as shown in Figure 1, barium ions are exposed on the 001 crystal face, which may make this face more reactive with other substances. Based on these characteristics, the 001 crystal face of barium sulfate was chosen as the representative surface for subsequent simulations and studies.

### 3.2. Adsorption Energy of Different Functional Groups on the Crystal Surface of Barium Sulfate 001

A comprehensive review of all the functional groups associated with scale inhibitors was undertaken. The results of the research indicate that compounds containing lone pairs of electrons are more effective in forming coordination complexes with the metal ions in scale. This interaction perturbs the precipitation equilibrium in the scale and shifts the dissolution equilibrium to the right, thereby effectively promoting scale’s dissolution. Common functional groups and their respective properties are listed in Table 2.

**Figure 2:**
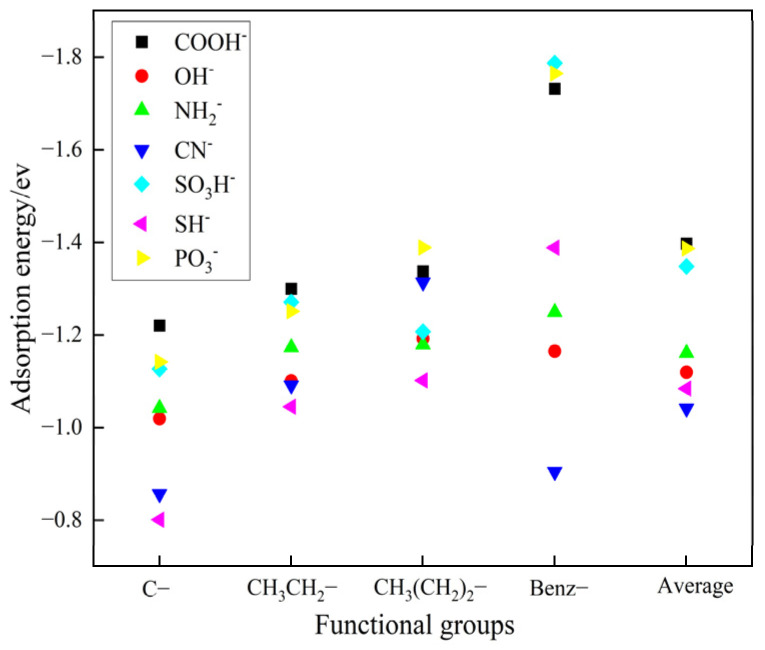
Adsorption energy of different functional groups on the crystal surface of barium sulfate 001.

As illustrated in Figure 2, the carboxylic acid group has a higher average adsorption energy compared to the other functional groups within the compounds. This observation is attributed to the effect of the side chain structure on the electronic environment, modulating the strength of electrostatic interactions and hydrogen bonding. In addition, as the side chain length increases, the adsorption energy of the carboxylic acid functional group also increases. The interaction between benzoic acid and the barium sulfate 001 surface shows the highest adsorption effect, with an energy of −1.7316 ev. This result suggests that hydrogen atoms are more effective than alkyl groups in reducing the electronic density of functional groups. Among these seven functional groups, carboxylic acids have the highest adsorption energy, followed by phosphonic acids [23,24]. This result indirectly confirms that modern chelating agents are predominantly composed of carboxylic and phosphonic acids. Given that carboxylic acids have the highest adsorption energy, we will prioritize the use of carboxylic acid functional groups in subsequent experimental designs.

### 3.3. Adsorption Energy of Azacrown Ethers

**Figure 3:**
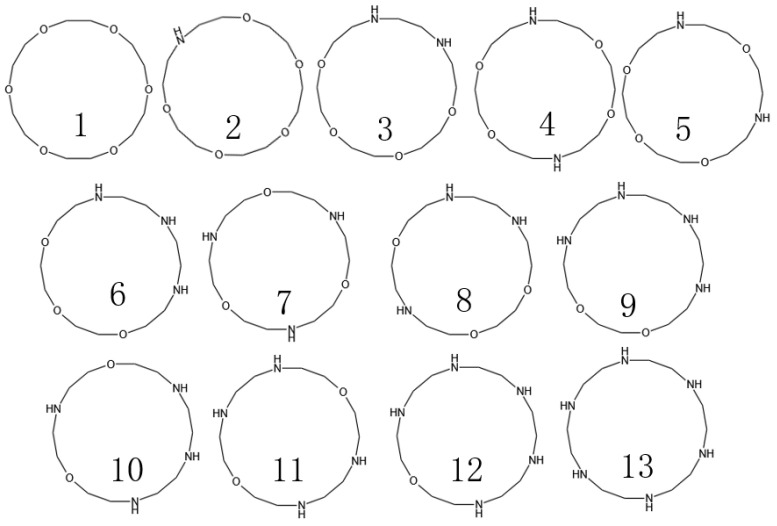
Design of azacrown ethers with different structures.

In previous studies, we successfully determined the interfacial properties of barium sulfate, as well as the major adsorption functional groups. Based on these findings, subsequently, we are focused on the number of nitrogen (N) and oxygen (O) atoms in the crown ether and their specific positions in the molecule. We found that 18-crown 6-ethers form the most stable complexes with Ba^2+^. However, they are less soluble in water and organic solvents. However, when nitrogen atoms were introduced into the crown ether structure as a medium-strength base to form azacrown ethers, their affinity to metal ions was significantly increased, and the rate of coordination was accelerated accordingly. Therefore, the structures of macrocyclic compounds were determined (Figure 3) by designing different amounts of N to replace the O atoms of 18-crown 6-ethers and calculating the adsorption energies for Ba^2+^ of the corresponding structures.

Among the many compounds, compounds **2**, **9**, and **8** exhibited the strongest adsorption properties. Although compounds **9** and **8** were outstanding in their adsorption capacity, they were synthesized at a high cost and in limited yields, so this study focused on compound **2**. The HOMO-LUMO energy gap value (ΔE) is a measure of the ability of electrons to jump from the highest occupied molecular orbital (HOMO) to the lowest unoccupied molecular orbital (LUMO), and this metric reflects the participation of molecules in the chemical reaction activity to a certain extent. A smaller value for the HOMO-LUMO energy gap implies that electron leaps are easier. However, according to the data in Table 3, the compounds with the lowest energy gap values do not necessarily have the highest Ba ion adsorption efficiency. Therefore, the main focus of this study is the adsorption energy, and the HOMO-LUMO energy gap value is used as a reference index.

### 3.4. Design of Linking Functional Groups

**Figure 4:**
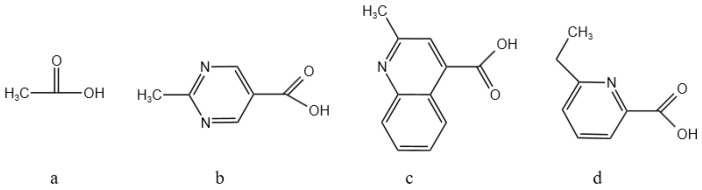
Design of different linking functional groups.

In order to increase the solubility of the macrocyclic compounds and to improve their adsorption of Ba ions, the functional groups were linked as shown in Figure 4: formic acid, pyridine carboxylic acid, quinoline carboxylic acid, and pyrimidine carboxylic acid corresponding to Figure 4a, Figure 4b, Figure 4c, and Figure 4d, respectively.

From Table 4, it can be seen that there is a significant difference in the adsorption capacity of different compounds for barium ions. Through a comparative analysis, the order of the adsorption energy of the different compounds for barium ions was obtained as **2d** > **2b** > **2c** > **2a**. That is, compound **2d** has the strongest adsorption capacity for barium ions, followed by compound **2b**. This finding suggests that the chemical structure and nature of the compounds have a direct influence. In particular, compound **2d** may have a stronger electron donor capacity or a more suitable spatial configuration, allowing it to form stable adsorption complexes with barium ions more efficiently. Compound **2a**, on the other hand, may have a relatively weak ability to adsorb barium ions due to limitations in its structure or electronic properties. This adsorption theory provides a theoretical basis for us to design new nitrogen-containing macrocyclic descaling agents.

### 3.5. Comparison Between New Macrocycle Descalers and Commonly Used Chelating Agents

Before investigating the performance of the new macrocycle scale inhibitors, their ecological properties were predicted using the ADMETlab 2.0 (accessed on 1 September 2024) and SwissADME programs (accessed on 12 September 2024) [25,26,27,28], and descriptors such as the ecological toxicity properties of the macrocycles were predicted and compared. The results in Table 5 show that the newly synthesized macrocycle scale inhibitors are non-carcinogenic, non-irritating to the skin, and non-irritating to the eyes. Macrocycle **2d** has a specific pKa value that allows it to dissociate H in aqueous solution and chelate with barium ions. Macrocycle **2d** substance has low water solubility due to the presence of aromatic rings in its structure.

### 3.6. Comparison of Their Mechanisms

**Figure 5:**
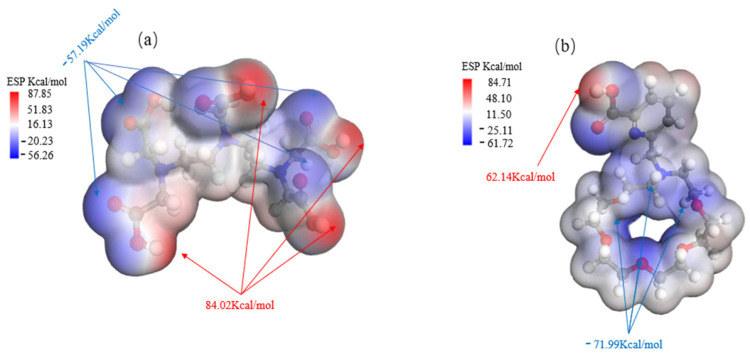
Comparison of ESP charts for DTPA (**a**) and macrocycle (**b**).

As demonstrated in Figure 5, the nucleophilic properties of DTPA are mainly due to the oxygen atoms within its carboxylic acid groups (-COOH), each of which carries lone pairs of electrons with an electrostatic potential of −57.19 kcal/mol. The availability of these lone electron pairs facilitates the formation of stable coordination bonds with metal ions.

In the macrocycle, the nucleophilic groups mainly consist of lone electron pairs on the nitrogen and oxygen atoms, which have an electrostatic potential of −71.99 kcal/mol. These lone pairs have the ability to form ligand bonds with metal ions or other electrophilic reagents. Additionally, the oxygen atoms in the carboxylic acid group (-COOH) exhibit nucleophilicity and can participate in chemical reactions as proton donors. This nucleophilicity allows effective recognition and binding with metal ions, such as barium ions.

**Figure 6:**
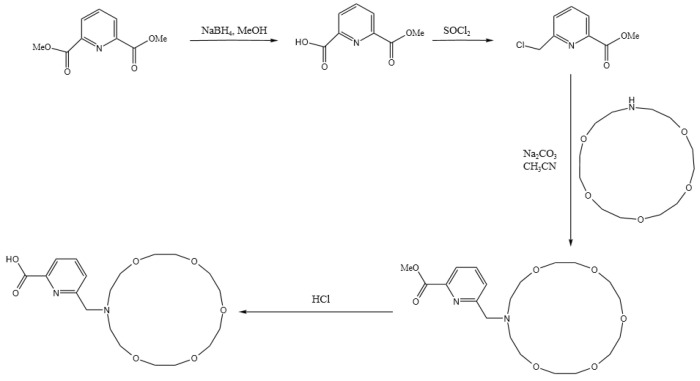
Macrocycle synthesis route.

As demonstrated by the mechanism in Table 6, the difference in their adsorption energy is particularly pronounced: the adsorption energy for Ba ions of DTPA is −5.6998 ev, while that of the macrocycle for Ba ions increases significantly to −9.8510 ev, representing a difference of −4.1512 ev. This difference is primarily due to the dominant role of electrostatic interactions, in which the oxygen atoms within the macrocycle interact with the metal ions robustly via electrostatic forces. These electrostatic forces arise mainly due to the charge attraction between the lone electron pairs of the oxygen atoms and the metal ions. During the coordination of the macrocycle with barium ions, electrostatic forces are the principal drivers of molecular recognition and complex formation. This is supported by the charge transfer data: the oxygen atoms in the macrocycle transfer more charge than those in DTPA, while the nitrogen atoms in DTPA show a slightly higher charge transfer than the nitrogen atoms in the macrocycle owing to van der Waals forces. The literature [29,30] meticulously outlines the synthetic pathways for macrocycles, as depicted in Figure 6. This comprehensive information is designed to offer chemical researchers clear and actionable guidance, facilitating efficient execution of their synthesis endeavors. Armed with these detailed synthetic routes, researchers are empowered to synthesize target compounds with greater speed and precision.

### 3.7. Molecular Dynamics

**Figure 7:**
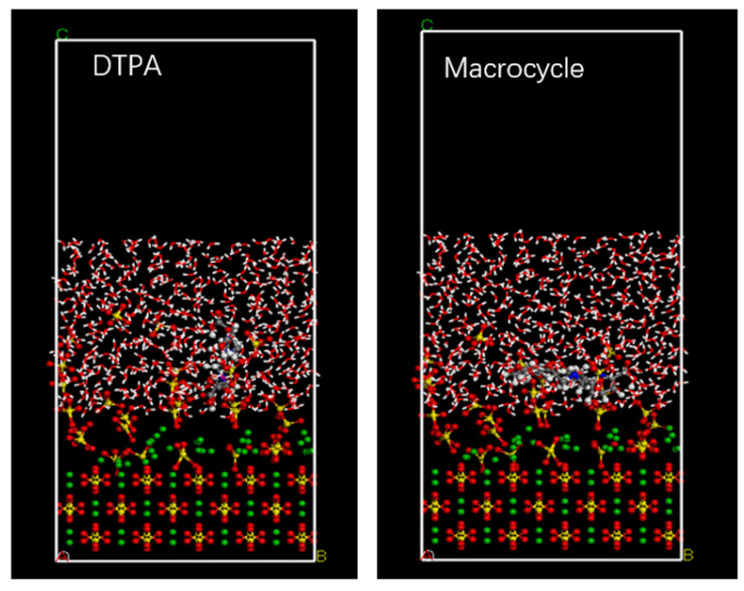
Molecular dynamics model.

**Figure 8:**
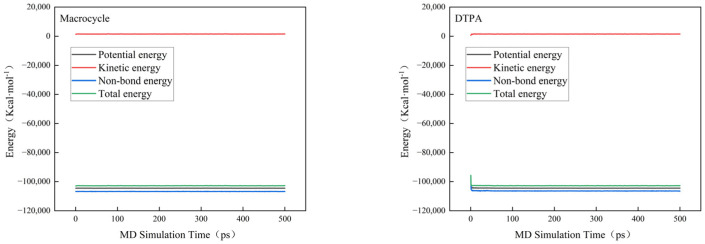
Energy changes in molecular dynamics.

In order to verify the efficacy of the macrocycle descaler, we performed molecular dynamics simulations at a temperature of 298 K. Figure 7 shows a model of the macrocycle’s reaction alongside DTPA, in which we introduced water molecules to simulate the actual descaling environment. The simulation lasted for 500 ps, during which we monitored the changes in the potential, non-bond, kinetic, and total energies and found that the errors in all of these energy parameters were less than 5% (as shown in Figure 8). Based on these data, we can assume that the system reached kinetic equilibrium.

**Figure 9:**
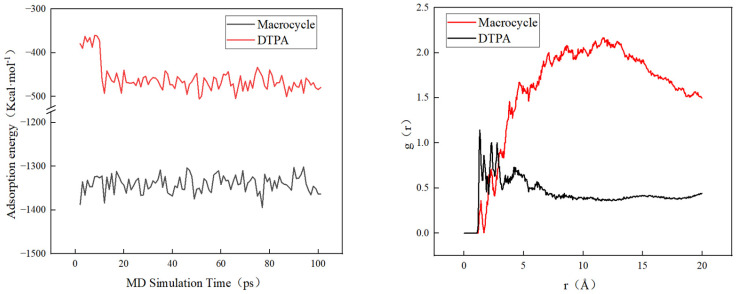
Adsorption energy changes and RDF in molecular dynamics.

Figure 9 shows that macrocycles have a significantly stronger adsorption capacity for barium sulfate scale than DTPA. Specifically, the average adsorption energy of macrocycles is three times higher than that of DTPA, which indicates that macrocycles are more effective in adsorbing and removing barium sulfate scale. In addition, radial distribution function (RDF) analysis showed that barium sulfate tended to aggregate more near the macrocycle and adsorbed over a shorter distance. This enhanced adsorption capacity was attributed to the macrocyclic effect of the macrocycle, which was able to interact more closely with the barium sulfate. In contrast, DTPA adsorbs primarily through the chelation of carboxylic acid groups, which results in a relatively long distance to the barium ions.

## 4. Conclusions

(1) By calculating the surface energy of different crystal facets of barium sulfate, we found that the 001 and 210 facets of barium sulfate have the lowest surface energy, indicating that these facets are the most structurally stable.
(2) After designing a variety of functional groups, azacrown ethers, and linking groups, we selected a macrocycle descaling agent and compared it with DTPA. This study’s results show that this new macrocycle descaling agent is safer in terms of its biological toxicity, and its adsorption energy for barium ions is significantly enhanced, reaching −9.8510 ev.
(3) The high adsorption capacity of the macrocycle descaling agent is mainly due to its strong electrostatic interaction. This electrostatic force is mainly due to the charge attraction between the lone pairs of electrons on the oxygen atoms and metal ions. During the coordination of the macrocycle with barium ions, electrostatic forces play a central role in molecular recognition and complex formation. This is further confirmed by the charge transfer data: the oxygen atoms in the macrocycle transfer more charge to the barium ions, while the nitrogen atoms in DTPA have a slightly higher charge transfer capability due to the influence of van der Waals forces.

## Figures and Tables

**Table 1:** Surface energy of barium sulfate.

Crystal	001	100	210	120	010	011	012	201	212
Surface energy (J·m^−2^)	0.0309	39.1026	0.0327	5.7746	0.0403	0.0984	0.0636	8.3453	5.5016

**Table 2:** Functional groups and their properties.

Functional Groups	Characteristic
COOH^−^	The hydrophilic group exhibits remarkable water solubility. The lone pairs of electrons on the oxygen atom allow for the formation of multidentate chelate rings with metal ions, which significantly increases ion solubility in water and enhances the capacity for scale removal.
OH^−^	The hydroxyl group (OH) is a hydrophilic moiety consisting of a negatively charged oxygen atom and a positively charged hydrogen atom. Consequently, molecules bearing hydroxyl groups typically exhibit high solubility in water.
NH_2_^−^	The amino group (NH_2_) is a hydrophilic functional group with lone pairs of electrons. The nitrogen atom within the amino group possesses an unshared electron pair, allowing it to form hydrogen bonds with water molecules, thereby conferring excellent water solubility. Furthermore, the nitrogen atom can establish coordination bonds with metal ions, making it widely applicable in metal ion capture and molecular interactions.
CN^−^	Exhibiting electrophilicity and chemical stability, it forms stable complexes with metal ions, making it highly effective for metal ion extraction.
SO_3_H^−^	The sulfonic acid group (SO_3_H) is a highly polar moiety with exceptional water solubility, readily dissolving in polar solvents. It features two π bonds and three negatively charged oxygen atoms, which impart a repulsive effect on cations. Additionally, it exhibits notable resistance to heat and salts.
SH^−^	The thiol group (SH) is hydrophilic and capable of forming stable complexes with metal ions, making it highly effective for the adsorption and removal of heavy metal ions.
PO_3_^−^	The phosphate group (PO_3_) is a hydrophilic moiety with lone electron pairs, capable of forming stable chelates with metal ions, thereby inhibiting the formation of scale in water systems. Compared to conventional chemical scale inhibitors, it offers superior biodegradability and reduced toxicity.

**Table 3:** Adsorption energy, HOMO, LUMO, and energy gap of different macrocyclic compounds for Ba^2+^.

Number	Adsorption Energy/ev	HOMO/ev	LUMO/ev	△E/ev
**1**	−8.3002	−5.3762	1.0159	−6.3921
**2**	−8.6259	−4.5514	1.0949	−5.6463
**3**	−8.5235	−4.3468	1.1655	−5.5124
**4**	−8.5142	−4.4664	1.0106	−5.4771
**5**	−8.5264	−4.2272	0.9971	−5.2243
**6**	−8.0783	−4.5303	1.0469	−5.5772
**7**	−8.2592	−4.6000	0.7375	−5.3375
**8**	−8.7164	−4.4747	0.8184	−5.2931
**9**	−8.8077	−4.5940	0.8439	−5.4379
**10**	−8.4440	−4.3927	1.1233	−5.5161
**11**	−8.2868	−4.3060	1.0813	−5.3873
**12**	−8.5840	−4.4030	0.9157	−5.3187
**13**	−8.5390	−4.5462	0.8724	−5.4186

**Table 4:** Comparison of adsorption energies of macrocyclic compound **2** and different linking groups with Ba^2+^.

Compound	Linking Functional Groups	Adsorption/ev
**2**	**a**	−8.4474
**2**	**b**	−9.7774
**2**	**c**	−9.1616
**2**	**d**	−9.8510

**Table 5:** Comparison of biological indicators between new macrocycle descalers and commonly used chelating agents.

Name	ΔE	Skin Corrosion	Eye Corrosion	Neurotoxicity	pKa	Water Solubility logS (ESOL)
**2d**	−1.8564	0.04	0.02	−2.55	4.86	−0.57 (Very soluble)
DOTA	3.0939	0.28	0.85 (toxic)	−1.95	6.24	4.85 (Highly soluble)
CDTA	3.2647	0.32	0.27	−1.83	6.47	1.54 (Highly soluble)
DTPA	3.1177	0.24	0.42	−1.91	7.23	4.15 (Highly soluble)
EGTA	3.7456	0.33	0.49	−1.91	5.68	2.82 (Highly soluble)
EDTA	3.4851	0.44	0.68	−1.78	5.41	2.78 (Highly soluble)

**Table 6:** Comparison of mechanisms of action.

Mechanisms	DTPA	Macrocycle	Difference
Adsorption/ev	−5.6998	−9.8510	−4.1512
O charge number transferred	−0.36	−0.39	−0.03
N charge number transferred	−0.29	−0.22	0.07
van der Waals/Kcal·mol^−1^	20.02	14.65	−5.37
Electrostatic/Kcal·mol^−1^	−85.20	−143.37	−58.17

## Data Availability

Data are contained within the article.
